# Emerging Fungal Pathogen Candida auris Evades Neutrophil Attack

**DOI:** 10.1128/mBio.01403-18

**Published:** 2018-08-21

**Authors:** Chad J. Johnson, J. Muse Davis, Anna Huttenlocher, John F. Kernien, Jeniel E. Nett

**Affiliations:** a Department of Medicine, University of Wisconsin, Madison, Wisconsin, USA; b Department of Pediatrics, University of Wisconsin, Madison, Wisconsin, USA; c Department of Medical Microbiology and Immunology, University of Wisconsin, Madison, Wisconsin, USA; Duke University Medical Center

**Keywords:** Candida auris, fungi, immune, neutrophil, neutrophil extracellular trap, zebrafish

## Abstract

Candida auris has recently emerged as the first fungal pathogen to cause a global public health threat. The reason this species is causing hospital-associated outbreaks of invasive candidiasis with high mortality is unknown. In this study, we examine the interaction of C. auris with neutrophils, leukocytes critical for control of invasive fungal infections. We show that human neutrophils do not effectively kill C. auris. Compared to Candida albicans, neutrophils poorly recruited to C. auris and failed to form neutrophil extracellular traps (NETs), which are structures of DNA, histones, and proteins with antimicrobial activity. In mixed cultures, neutrophils preferentially engaged and killed C. albicans over C. auris. Imaging of neutrophils in a zebrafish larval model of invasive candidiasis revealed the recruitment of approximately 50% fewer neutrophils in response to C. auris compared to C. albicans. Upon encounter with C. albicans in the zebrafish hindbrain, neutrophils produced clouds of histones, suggesting the formation of NETs. These structures were not observed in C. auris infection. Evasion of neutrophil attack and innate immunity offers an explanation for the virulence of this pathogen.

## IMPORTANCE

The emerging fungal pathogen Candida auris has produced numerous outbreaks of invasive disease in hospitals worldwide. Why this species causes deadly disease is unknown. Our findings reveal a failure of neutrophils to kill C. auris compared to the most commonly encountered *Candida* species, C. albicans. While neutrophils produce neutrophil extracellular traps (NETs) upon encounter with C. albicans, these antimicrobial structures are not formed in response to C. auris. Using human neutrophils and a zebrafish model of invasive candidiasis, we show that C. auris poorly recruits neutrophils and evades immune attack. Identification of this impaired innate immune response to C. auris sheds light on the dismal outcomes for patients with invasive disease.

## OBSERVATION

Candida auris, an emerging fungal pathogen, causes nosocomial outbreaks of invasive candidiasis with mortality rates approaching 60% (1–3). Little is known about the pathogenesis of this species that has arisen in the last 10 years, and it is unclear why it is rapidly spreading in hospitals worldwide (4). C. auris is the first fungal pathogen labeled as a public health threat due to its ability to readily colonize skin, spread efficiently person to person, and cause severe disease with high fatality (5). Drug resistance is common for this species, with the vast majority of isolates exhibiting fluconazole resistance and nearly half demonstrating resistance to two or more antifungal drug classes (1). Beyond this obstacle to treatment, mortality rates are high even for patients treated with appropriate antifungals (1).

Neutrophils are critical for control of numerous invasive fungal infections, including candidiasis (6, 7). These immune cells kill fungi through phagocytosis or the release of neutrophil extracellular traps (NETs), which are structures of DNA, histones, and proteins with antimicrobial activity (8–10). However, unlike many fungal infections, neutropenia has not been reported as a common risk factor for C. auris infection (1, 11). This observation prompted us to explore the interaction of neutrophils with C. auris to account for the unexplained virulence of this emerging pathogen.

**FIG 1:**
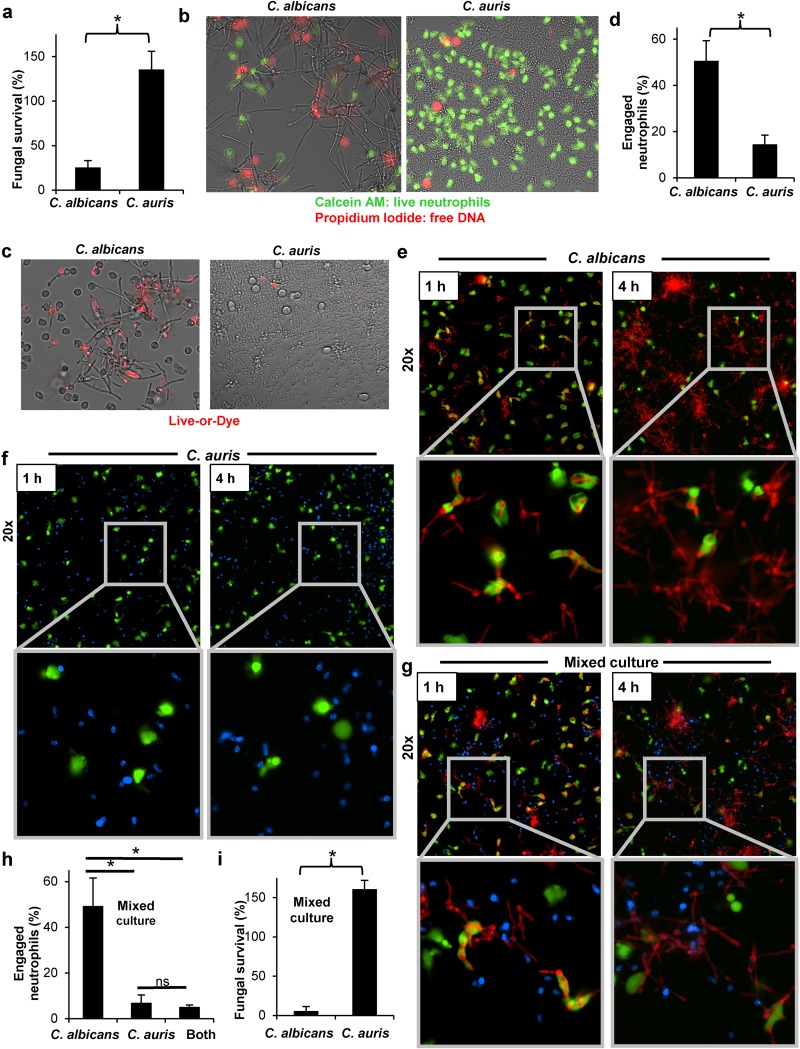
C. auris resists neutrophil attack. (a) Human neutrophils were cocultured with C. albicans or C. auris for 4 h, and fungal survival was measured by plate counts (mean with standard error of the mean [SEM] shown; *, *P* < 0.05 by Student’s *t* test). (b) C. albicans or C. auris cells were cocultured with calcein AM-labeled human neutrophils (green) for 4 h, and samples were imaged following propidium iodide (red) staining of free DNA and cells with membrane disruption. (c) Following a 4-h coculture with human neutrophils, *Candida* viability was accessed by the Live-or-Dye detection of dead cells (red). (d to f) RFP-tagged C. albicans (red) or calcofluor white-stained C. auris (blue) were cocultured with calcein AM-labeled human neutrophils (green) and imaged at 1 and 4 h. The number of neutrophils engaging C. albicans or C. auris at 1 h were enumerated (*n =* 3; mean with SEM shown; *, *P* < 0.05 by Student’s *t* test) (d), and images are shown for C. albicans (e) and C. auris (f). (g and h) Calcein AM-labeled human neutrophils (green) were added to a mixture of C. albicans (red) and C. auris (blue), both at 50% of the concentrations in panels a and d and imaged after 1 and 4 h. Neutrophils associating with C. albicans or C. auris in the mixed culture were enumerated (*n =* 3; mean with SEM shown; *, *P* < 0.05 by analysis of variance [ANOVA] with Holm-Sidak pairwise comparisons [h]), and images are shown (g). (i) Human neutrophils were cocultured with mixture of C. albicans and C. auris cells for 4 h, and fungal survival was measured by plate counts (*n =* 3; mean with SEM shown *, *P* < 0.05 by Student’s *t* test).

To determine if neutrophils effectively eradicate C. auris, we measured fungal viability after encounter with human neutrophils and included C. albicans, the most commonly isolated *Candida* species, for comparison (Fig. 1a). After a 4-h coculture, neutrophils inhibited C. albicans growth by 75%. In stark contrast, the burden of C. auris was not impacted and C. auris replicated beyond the initial inoculum. Evaluation of fungal death by either propidium iodide or Live-or-Dye staining similarly revealed very little killing of C. auris by neutrophils (Fig. 1b and c).

We next theorized that C. auris may resist neutrophil killing by avoiding effective engagement and phagocytosis. Using fluorescence microscopy, we analyzed neutrophil-*Candida* interactions (Fig. 1d to f). At 1 h, very few neutrophils (15%) were either engulfing or adherent to coincubated C. auris cells. In contrast, 50% of neutrophils were associated with C. albicans. We next performed a competition experiment and exposed neutrophils to a mixture containing both *Candida* species. The preference of human neutrophils for C. albicans over C. auris was even more apparent for the mixed culture (Fig. 1g and h; see Movie S1 in the supplemental material). Approximately half of the neutrophils associated with C. albicans, while C. auris appeared to be ignored, engaged by fewer than 10% of the neutrophils. To assess if the more robust recruitment and engagement of neutrophils by C. albicans correlated with fungal killing, we coincubated neutrophils with a mixed culture and measured fungal viability. Neutrophils exhibited a potent antifungal response to C. albicans, with only 5% surviving, while C. auris was strikingly resistant to neutrophil killing (Fig. 1i).

10.1128/mBio.01403-18.3 MOVIE S1  Human neutrophil interactions with C. albicans and C. auris in a mixed culture. Calcein AM-labeled neutrophils (green) were added to culture containing half C. albicans cells (RFP [red]) and half C. auris cells (calcofluor white [blue]). Interactions were cocultured with calcein AM-labeled human neutrophils (green) and imaged at 30 min and 4 h. Images were collected at 1-min intervals from 50 to 170 min. The video is shown at 5 frames per s. Download MOVIE S1, AVI file, 6.1 MB. Copyright © 2018 Johnson et al. 2018 Johnson et al. This content is distributed under the terms of the Creative Commons Attribution 4.0 International license.

Upon examination of the neutrophils in coculture with C. albicans or C. auris over time, we were struck by the difference in neutrophil viability (Fig. 1e and f; see Fig. S1 in the supplemental material). After 4 h, the viability of neutrophils cocultured with C. albicans decreased by half, as evidenced by loss of the fluorescent calcein AM stain. Remarkably, upon exposure to C. auris, nearly all neutrophils remained viable. A similar pattern of neutrophil death was apparent with propidium iodide staining as well (Fig. 1b). As C. albicans is known to induce the formation of NETs, we suspected that neutrophils may be undergoing NETosis, the associated programmed cell death (8). This finding suggested that C. auris may be limiting NET formation.

10.1128/mBio.01403-18.1 FIG S1  Viability of neutrophils exposed to *Candida*. (a) C. albicans (RFP [red]) or C. auris (calcofluor white [blue]) cells were cocultured with calcein AM-labeled human neutrophils (green) and imaged at 30 min and 4 h. (b) The viability of neutrophils exposed to *Candida* was assessed based on retention of calcein AM stain. *n =* 3; mean with SEM shown; *, *P* < 0.05 by Student’s *t* test. Download FIG S1, PDF file, 0.4 MB. Copyright © 2018 Johnson et al. 2018 Johnson et al. This content is distributed under the terms of the Creative Commons Attribution 4.0 International license.

**FIG 2:**
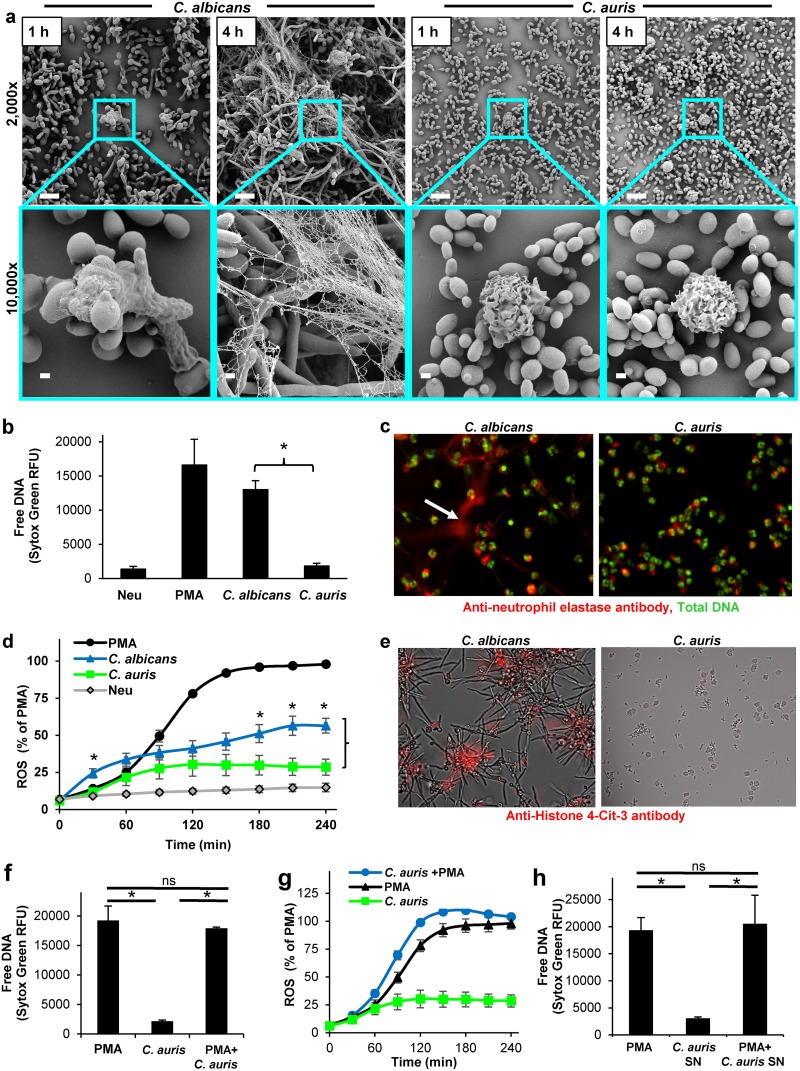
C. auris fails to trigger the formation of NETs *in vitro*. (a) C. albicans or C. auris were cocultured with human neutrophils and imaged by scanning electron microscopy after 1 and 4 h. The measurement bars represent 10 and 1 µm for the 2,000× and 10,000× images, respectively. (b) Human neutrophils were exposed to *Candida* for 4 h, and NET release was estimated by Sytox green detection of free DNA. Data from 4 experiments performed in triplicate were combined. Neutrophil responses to C. albicans and C. auris were analyzed by Student’s *t* test. *, *P* < 0.05; mean with SEM shown. (c) Neutrophils were cocultured with *Candida* for 4 h, fixed, immunolabeled with anti-neutrophil elastase (red) and for total DNA with Sytox green (green), and imaged. The arrow highlights extracellular neutrophil elastase. (d) Production of ROS in response to *Candida* was measured by fluorescence after neutrophils were prestained with the oxidative stress indicator chloromethyl 2′,7′-dichlorofluorescein (CM-DCF). The mean and SEM of 6 experiments performed in triplicate are shown. Data for C. albicans and C. auris at each time point were analyzed by Student’s *t* test (*, *P* < 0.05). (e) Neutrophils were cocultured with *Candida* for 4 h, fixed, immunolabeled with an anti-citrullinated H4 antibody (red), and imaged. (f) C. auris was cocultured with human neutrophils in the presence and absence of PMA (100 nM) for 4 h, and NET release was estimated by Sytox green detection of free DNA. Experiments were performed in triplicate on 3 occasions (mean with SEM shown). Data were analyzed by ANOVA with pairwise comparisons using the Holm-Sidak method (*, *P* < 0.05; ns, not significant). (g) Production of ROS in response to C. auris was measured by fluorescence after neutrophils were prestained with oxidative stress indicator CM-DCF and cocultured with C. auris over 4 h. Experiments were performed in triplicate on 3 occasions (mean with SEM shown). (h) Supernatants (SN) were collected from C. auris cultures propagating for 4 h. The impact of supernatants on PMA-induced NET release was assessed by Sytox green. Experiments were performed in triplicate on 3 occasions, and data were analyzed by ANOVA with pairwise comparisons using the Holm-Sidak method (mean with SEM shown; *, *P* < 0.05). Neu, neutrophil-only control; PMA, phorbol 12-myristate 13-acetate.

To examine NET formation in response to C. auris, we utilized scanning electron microscopy (Fig. 2a). Imaging revealed the phagocytosis of C. albicans at 1 h, followed by the formation of web-like structures by 4 h, indicating the release of NETs, as previously described (8). In contrast, neutrophils appeared rounded upon encounter with C. auris at both 1 and 4 h. Neutrophils engaging in phagocytosis or releasing NETs were rarely observed. To quantify NET formation, we measured free DNA with Sytox green (Fig. 2b). Following a 4-h exposure to C. albicans, neutrophils released high levels of free DNA, similar to those treated with phorbol 12-myristate 13-acetate (PMA), a potent inducer of NETs. However, C. auris did not trigger free DNA release, consistent with the lack of NET production observed in microscopy experiments.

Our next experiments were designed to dissect the influence of C. auris on specific molecular aspects of NET formation. We utilized immunofluorescence microscopy to track neutrophil elastase, a granular protein that translocates to the nucleus, cleaves histones, and is then released extracellularly during NET formation (12, 13). In response to C. albicans, neutrophil elastase was detected extracellularly, colocalizing with the DNA fibers of NETs (Fig. 2c). However, extracellular release of neutrophil elastase was not observed in response to C. auris. The generation of ROS, a process integral to the translocation of neutrophil elastase and subsequent NET formation, was also blunted in response to C. auris (Fig. 2d). We next examined citrullination, the enzymatic conversion of arginine residues to citrulline, which occurs on histones during NET formation (14) (Fig. 2e). While C. albicans triggered the release of citrullinated histones, C. auris did not. Even intracellular histone citrullination, signifying an early stage in NET formation, was not observed in response to C. auris.

We also considered the possibility that C. auris may broadly inhibit NET formation and associated reactive oxygen species (ROS) production by neutrophils. To test this hypothesis, we examined the neutrophil response to C. auris in the presence of a strong NET-inducing stimulus. C. auris did not inhibit PMA-induced NET formation or ROS production (Fig. 2f and g). Similarly, supernatants produced by C. auris failed to inhibit PMA-induced NET formation (Fig. 2h). We concluded that C. auris was not actively inhibiting NET formation and ROS production, but rather evaded these defense responses. These findings are consistent with the mixing studies showing C. auris did not impair the activity of neutrophils against C. albicans (Fig. 1g to i).

**FIG 3:**
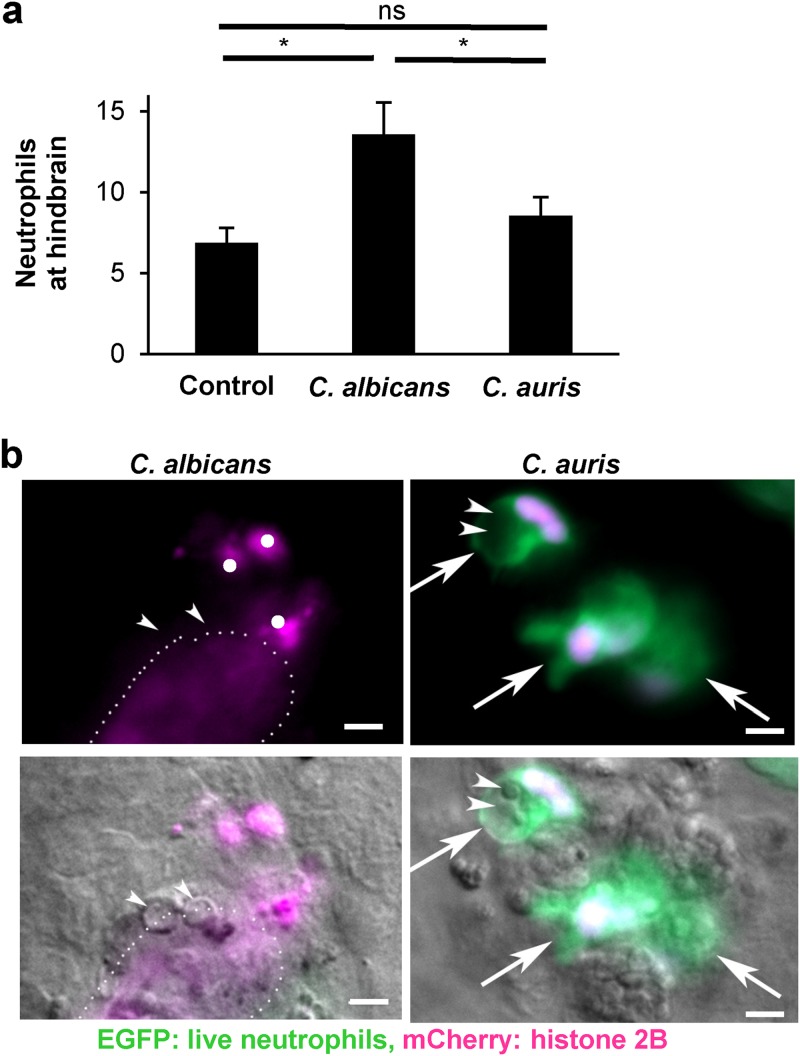
Neutrophil-*Candida* interactions in zebrafish. (a) Zebrafish larvae were infected with *Candida* by hindbrain injection and neutrophil recruitment to the hindbrain was assessed after 4 h. Experiments were performed on 3 occasions with at least 10 larvae per condition, and data were analyzed by ANOVA with pairwise comparisons using the Holm-Sidak method (*, *P* < 0.05; ns, not significant). (b) Transgenic larvae with neutrophils expressing cytosolic EGFP (green) and mCherry-tagged histone 2B (red) were infected with *Candida* by hindbrain injection and imaged after 24 h. Arrowheads indicate yeast cells. Arrows indicate intact neutrophils. Single dots show condensed nuclear material consistent with dead neutrophil fragments. The dotted line outlines the cloud of tagged histone signal. The measurement bars represent 5 µm.

To explore the clinical relevance of neutrophil interactions with C. auris *in vivo*, we employed a zebrafish model of invasive candidiasis, as the translucent larvae are ideal for the tracking and imaging of leukocytes (15). Following hindbrain inoculation, neutrophils were readily recruited to C. albicans by 4 h, as described previously (15) (Fig. 3a). In contrast, C. auris recruited few neutrophils, exhibiting neutrophil recruitment similar to the saline-injected controls. To examine the neutrophil response to *Candida in vivo*, we utilized a transgenic zebrafish line with neutrophil-specific enhanced green fluorescent protein (EGFP) expression in the cytoplasm as well as neutrophil-specific mCherry tagged to histone H2 localized to nuclear material. By 24 h after inoculation of C. albicans into the hindbrain, a subset of neutrophils produced clouds of tagged histones (Fig. 3b). These appeared in close proximity to yeast, which may have been previously ingested. The extracellular release of the structures is most consistent with the formation of extracellular traps with histone studding of DNA. Furthermore, as the EGFP is soluble in the cytoplasm, the loss of EGFP signal in these neutrophils signifies membrane permeability and cell death. In contrast, the majority of the neutrophils of C. auris-infected zebrafish remained viable with the tagged histone colocalizing in the nucleus, and NET-like structures were not observed.

In summary, our studies show vastly different responses by neutrophils to C. auris and C. albicans. C. auris resists killing by human neutrophils. Upon encounter, neutrophils do not effectively engage C. auris and pathways for NET formation that are critical for host elimination of this pathogen are not triggered. Competition experiments reveal a strong preference of human neutrophils for engagement of C. albicans over C. auris, ultimately leading to 10-fold-higher inhibition of C. albicans over C. auris. A zebrafish larva infection model similarly uncovered a failure of C. auris to recruit neutrophils. We propose that this altered innate immune response contributes to the poor outcomes observed in patients with invasive C. auris infection, by allowing this species to go unrecognized by neutrophils.

Using murine models of invasive candidiasis, several groups have found C. auris to be less virulent than C. albicans, prompting the use of immunocompetent models (16, 17). It is unclear why the mice appear to control C. auris, while human neutrophils fail to recognize and kill the pathogen. One explanation may be the species difference in neutrophil receptors important for the killing of *Candida* (18, 19). In addition, virulence may vary by C. auris strain. For example, an evaluation of a collection of isolates by Borman et al. revealed strain-specific virulence in the Galleria mellonella model, with the most virulent C. auris strains exhibiting pathogenicity similar to that of C. albicans (20).

C. auris genotypes vary based on geography and cluster into clades (1). The C. auris strain used in the present study was isolated from a patient in India and phylogenetically placed in the South Asian or India/Pakistan clade. We selected a strain from this clade to investigate, as recent nosocomial outbreaks in both the United Kingdom and the United States have been caused by members of the South Asian clade (21, 22). However, given the genetic diversity between clades, examination of the neutrophil response to C. auris strains belonging to other clades would be of great interest.

As C. auris has only recently emerged, very little is known about virulence factors that may influence interaction with the host. Unraveling the molecular mechanisms impeding the engagement and activation of neutrophils may not only offer an explanation for the virulence of this pathogen, but also provide avenues to the development of therapeutics for this emerging global health threat.

### Organisms and inoculum.

C. auris (B11203), C. albicans SC5314, and red fluorescent protein (RFP)-labeled C. albicans CA14 mCherry were utilized (1, 23). Cultures were propagated overnight in yeast extract-peptone-dextrose (YPD) supplemented with uridine at 30°C on an orbital shaker at 200 rpm, diluted 1:20 in YPD, propagated for 2 h, and washed and resuspended in Dulbecco’s phosphate-buffered saline (DPBS).

### Human neutrophil collection.

Blood was obtained from volunteer donors with written informed consent through a protocol approved by the University of Wisconsin Internal Review Board. Neutrophils were isolated with MACSxpress neutrophil isolation and MACSxpress erythrocyte depletion kits (Miltenyi Biotec, Inc., Auburn, CA) and suspended in RPMI 1640 (without phenol red) supplemented with 2% heat-inactivated fetal bovine serum (FBS) and supplemented with glutamine (0.3 mg/ml). Incubations were at 37°C with 5% CO_2_.

### Sytox green assays.

*Candida* (2 × 10^5^ cells/96-well plate) and neutrophils (2 × 10^5^ cells/96-well plate) were cocultured and free DNA was measured by fluorescence (500/528 nm) after the addition of Sytox green, as previously described (24, 25). For a subset of experiments, phorbol myristate acetate (PMA [100 nM]) was included. Supernatants for *Candida* (3 × 10^7^ cells/ml) growing in RPMI for 4 h were centrifuged at 1,200 × *g* at 4°C for 10 min and filter sterilized.

### Measurement of ROS.

Neutrophils were stained with the fluorescent oxidative stress dye CMH (2) CFDA (Life Technologies, Inc., Eugene, OR) in DPBS for 10 min in the dark and added to *Candida* plates (2 × 10^5^ cells/96-well plate) at 2 × 10^5^ neutrophils/96-well plate as previously described (25). Fluorescence (495/527 nm) was recorded. For a subset of experiments, PMA (100 nM) was included. Background fluorescence was determined for each condition and subtracted from total fluorescence values prior to data analysis.

### Killing assays.

Neutrophils (1 × 10^7^ cells/12-well plate) were cocultured with *Candida* cells (2 × 10^7^ cells/12-well plate) for 4 h. Cultures were collected using cell scrapers, and neutrophils were lysed in double-distilled H_2_O (ddH_2_O) in the presence of DNase (1 U/ml [Sigma]) for 40 min. Serial dilutions were plated on YPD agar for assessment of viability. For studies involving the combination of C. albicans and C. auris, 1 × 10^7^ cells/well of each species were incubated to maintain an overall 1:2 effector/target ratio. Dilutions were plated on CHROMagar to distinguish species.

### Fluorescence imaging *in vitro.*

For fluorescent imaging, *Candida* cells (2 × 10^5^ cells/well) were loaded into the wells of a tissue culture-treated μ-Slide (8 wells). For a subset of experiments, *Candida* was preincubated with calcofluor white (100 µg/ml) for 10 min in the dark at room temperature and washed 3 times with DPBS. Neutrophils, stained with calcein AM (Thermo, Fisher Scientific, Waltham, MA) at 0.5 µg/ml in DPBS for 10 min at room temperature in the dark, were added at a concentration of 2 × 10^5^ cells/well. RFP-labeled C. albicans CA14 mCherry was utilized to distinguish C. albicans from calcofluor white-stained C. auris. Neutrophil interactions were similar for RFP-labeled and calcofluor white-labeled C. albicans (see Fig. S2 in the supplemental material). *Candida*-neutrophil interactions were analyzed by time-lapse microscopy at 37°C with 5% CO_2_. Images were obtained every 60 s using a Nikon eclipse-TI2 inverted microscope equipped with a TI2-S-SS-E motorized stage, ORCA-Flash 4.0 LT sCMOS camera, stage top TIZW series Neco incubation system (Tokai Hit) and NIS elements imaging software. The video is shown at 5 frames per s. To determine the percentage of viable neutrophils, the number of neutrophils retaining the calcein AM stain in the field of view after 4 h was divided by the calcien A-labeled neutrophils at 30 min. The membrane-impermeable dye propidium iodide (3 µM) incubated with samples for 15 min at 37°C was used to visualize extracellular DNA (and disrupted membrane). For immunofluorescence imaging, *Candida* cells and neutrophils were cocultured for 4 h and fixed with 4% formaldehyde in DPBS for an additional 4 h. Samples were then immunolabeled with primary antibodies (anti-histone H4, citrulline 3, or anti-neutrophil elastase) as previously described (25). Sytox green (1 µM) was used to stain total DNA in membrane-permeable samples.

10.1128/mBio.01403-18.2 FIG S2  Neutrophil interactions with C. albicans strains. (a) Calcofluor white-stained C. albicans cells (blue) were cocultured with calcein AM-labeled human neutrophils (green) and imaged at 1 h. (b) RFP-tagged C. albicans cells (red) were cocultured with calcein AM-labeled human neutrophils (green) and imaged at 1 h. Download FIG S2, PDF file, 0.2 MB. Copyright © 2018 Johnson et al. 2018 Johnson et al. This content is distributed under the terms of the Creative Commons Attribution 4.0 International license.

### Scanning electron microscopy.

*Candida-*neutrophil interactions were examined by scanning electron microscopy as previously described (9, 25). Briefly, C. albicans and C. auris were cocultured with neutrophils for 1 or 4 h, after which samples were washed with DPBS, fixed overnight (4% formaldehyde, 1% glutaraldehyde, in PBS), washed with PBS, treated with 1% osmium tetroxide, and then washed again. Samples were dehydrated through a series of ethanol washes followed by critical point drying and subsequently mounted on aluminum stubs. Following sputter coating with platinum, samples were imaged in a scanning electron microscope (LEO 1530) at 3 kV.

### Zebrafish.

All animal procedures were approved by the Institutional Animal Care and Use Committee at the University of Wisconsin according to the guidelines of the Animal Welfare Act, and The Institute of Laboratory Animal Resources *Guide for the Care and Use of Laboratory Animals*. A zebrafish larval hindbrain infection model of invasive candidiasis was utilized to assess neutrophil recruitment, as described previously (15). Larval zebrafish were incubated at 28.5°C in E3 buffer and manually dechorionated between 24 and 30 hpi. Larvae were anesthetized in 0.2 mg/ml tricaine (ethyl 3-aminobenzoate; Sigma-Aldrich). Microinjection was performed as previously described (26), with the alteration that larvae were positioned on a 3% agarose plate formed with holding grooves as previously described elsewhere (27). For quantification of neutrophil recruitment, 3 to 5 nl of C. albicans or C. auris at a concentration of 1 × 10^7^ CFU/ml in PBS was injected in the hindbrain of transgenic zebrafish with EGFP expressed under control of the *lycZ* promoter in neutrophils [*Tg*(*lyz*::*EGFP*), also known as nz117Tg] (28). After a 4-h incubation at 28.5°C, fish were placed in fixation buffer {0.1 M PIPES [piperazine-*N*,*N*′-bis(2-ethanesulfonic acid)], 1 mM MgSO_4_, 2 mM EGTA, 4% formaldehyde} at 4°C overnight and imaged by fluorescence microscopy. For imaging of neutrophil responses and evaluation of NETs, adult *Tg*(*lyz*::*EGFP*) fish were crossed with another line expressing mCherry-tagged histone 2B in neutrophils *Tg*(*lyz*::*H2B-mCherry*) (29). The resulting larvae were similarly infected with *Candida* by hindbrain injection and imaged after 24 h of differential inference contrast (DIC)/epifluorescence imaging was performed on a Zeiss Z1 Observer-based system run with the Zeiss Zen software using a Zeiss EC Plan-Neofluar objective (40×/0.75 NA). Epifluorescence images were collected using a Photometrics Coolsnap ES^2^ camera. Live imaging of zebrafish larvae was performed with larvae anesthetized in tricaine as previously described (26) and simply resting on the bottom of a glass bottom dish or immobilized in 1% low-melt agarose.
