# Supplementary figures and images for: Emerging Fungal Pathogen Candida auris Evades Neutrophil Attack
Source: mBio. 2018 Aug 21;9(4):e01403-18. doi: 10.1128/mBio.01403-18 (PMC6106086; doi:10.1128/mBio.01403-18)

**a**

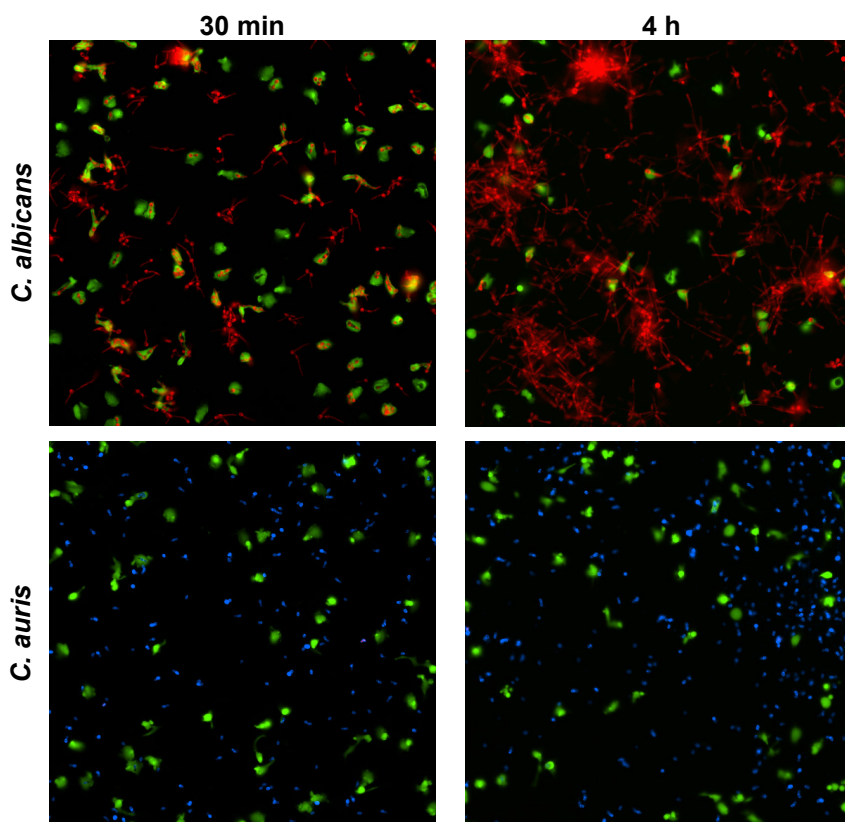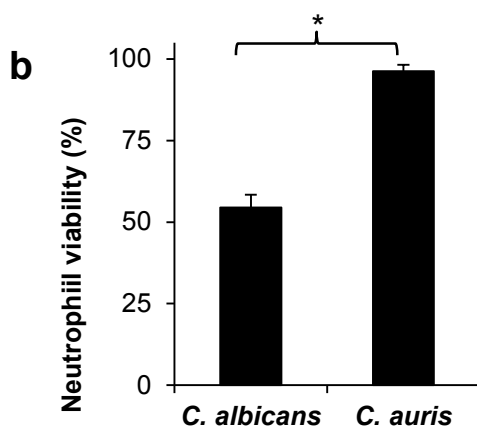

Supplement: FIG S1 [file mbo004184032sf1.pdf]

**a**

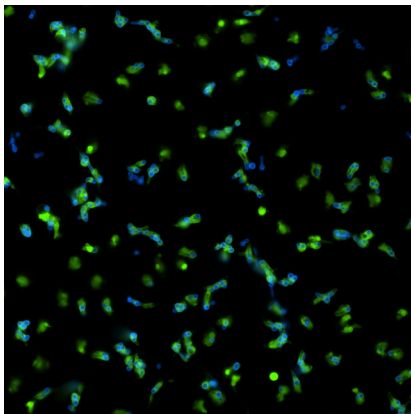

**b**

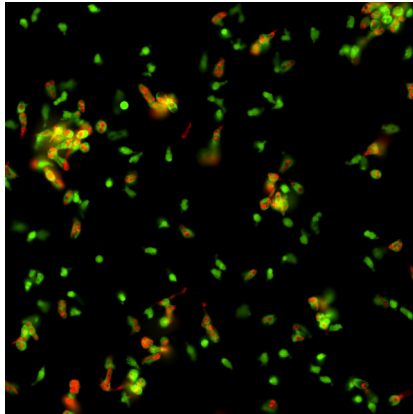

Supplement: FIG S2 [file mbo004184032sf2.pdf]
